# Climate change and species facilitation affect the recruitment of macroalgal marine forests

**DOI:** 10.1038/s41598-022-22845-2

**Published:** 2022-10-27

**Authors:** Margalida Monserrat, Steeve Comeau, Jana Verdura, Samir Alliouane, Guillaume Spennato, Fabrice Priouzeau, Gilbers Romero, Luisa Mangialajo

**Affiliations:** 1 Université Côte d’Azur, CNRS, ECOSEAS, Nice, France; 2 Sorbonne Université, CNRS, Laboratoire d’Océanographie de Villefranche, Villefranche-sur-Mer, France

**Keywords:** Climate-change ecology, Ecosystem ecology, Ecosystem services, Environmental sciences, Ocean sciences

## Abstract

Marine forests are shrinking globally due to several anthropogenic impacts including climate change. Forest-forming macroalgae, such as *Cystoseira* *s.l.* species, can be particularly sensitive to environmental conditions (e.g. temperature increase, pollution or sedimentation), especially during early life stages. However, not much is known about their response to the interactive effects of ocean warming (OW) and acidification (OA). These drivers can also affect the performance and survival of crustose coralline algae, which are associated understory species likely playing a role in the recruitment of later successional species such as forest-forming macroalgae. We tested the interactive effects of elevated temperature, low pH and species facilitation on the recruitment of *Cystoseira compressa*. We demonstrate that the interactive effects of OW and OA negatively affect the recruitment of *C. compressa* and its associated coralline algae *Neogoniolithon brassica-florida*. The density of recruits was lower under the combinations OW and OA, while the size was negatively affected by the temperature increase but positively affected by the low pH. The results from this study show that the interactive effects of climate change and the presence of crustose coralline algae can have a negative impact on the recruitment of *Cystoseira s.l.* species. While new restoration techniques recently opened the door to marine forest restoration, our results show that the interactions of multiple drivers and species interactions have to be considered to achieve long-term population sustainability.

## Introduction

The ocean plays an important role in the regulation of climate and offers numerous ecosystem services for humans. However, the ocean is affected by multiple anthropogenic impacts including climate change^1^. Ocean warming (OW) and ocean acidification (OA) are expected to affect most marine ecosystems with consequences to humans^2–4^. Ecosystems all around the globe are expected to experience reductions in habitat structure, biodiversity and trophic complexity as sea temperature rises^5^ and oceanic pH decreases^6,7^. At the same time, an expansion of opportunistic and turf-forming species is foreseen, with the consequent loss of ecosystem services^8–10^.

Large brown forest-forming macroalgae (which include the orders Laminariales, Tylopteridales, Desmarestiales, and Fucales) are dominant foundation species on intertidal and subtidal rocky shores in temperate and cold regions^11^. They form what is known as marine forests, which^12,13^ provide important ecosystem functions^14–16^. However, marine forests are shrinking globally^17,18^ due to several impacts such as urbanisation, marine farming, local pollution and herbivory^11,19,20^; making these habitats more sensitive to global change^5,21^. The structure and productivity of marine forests are influenced by many environmental factors that drive the growth, survival, reproduction and metabolism of the organisms, which in turn affect the whole habitat or ecosystem^22–24^. Global change effects on marine forests can vary according to the location, the population characteristics and the species^20,25,26^. As a result, in several cases, marine forests are constrained to locations with the most favourable conditions which could act as a refuge^26–28^. There is evidence that early life stages of these species are more vulnerable than adults which could lead, in the long term, to the loss of marine forests^12,29,30^. A high mortality rate is naturally observed during the early stages and the resilience of a population to future impacts can be largely dependent on efficient recruitment and development of juveniles^31,32^.

The shift in carbonate chemistry associated with OA causes an increase in dissolved CO_2_ that could favour photosynthesis and then the growth of photosynthetic organisms^6,33^. The increase in CO_2_ modifies the dissolved CO_2_ to O_2_ ratio at the RuBisCO active site, i.e., the key enzyme in carbon fixation metabolism. Because the latter emerged in an oxygen poor environment, it is characterised by a higher affinity for O_2_ than CO_2_^34^. Therefore, the current increase in dissolved CO_2_ to O_2_ ratio favours RuBisCO carbon fixation efficiency and then can favour the growth of photosynthetic organisms. To increase the RuBisCO carbon fixation efficiency, many algae also developed carbon concentration mechanisms (CCM), that increase the CO_2_ to O_2_ ratio in front of the RuBisCO fixation site^6,35^. Still, despite having or not having CCM it is not clear if most algae respond positively to an increase of CO_2_^6,35,36^. OA might, therefore, have beneficial effects for some species like large brown forest-forming macroalgae, that are thought to thrive at high CO_2_ concentrations^6,36,37^. However, calcifying organisms (e.g. foundation species like corals and coralline algae) are expected to be particularly affected by OA^38^. In particular, crustose coralline algae, which are important components of the understory of marine forests, are among the organisms potentially the most susceptible to OA^39,40^. This species can be directly and indirectly impacted by OA due to reduced calcification rates and increasing competition with algae which benefit from elevated CO_2_^35,41,42^. Crustose coralline algae are among the first colonizers of bare rock on euphotic marine habitats and are quickly overgrown by later successional species such as the more structurally complex large brown forest-forming macroalgae^43,44^. Some authors^45^ report that crustose coralline algae could help in the maintenance of alternative habitat states by preventing the recruitment of later colonizers (e.g. large brown macroalgae), even if this could be species-specific^46^. While other studies suggest that crustose coralline algae could, in contrast, enhance biodiversity by facilitating the settlement of later colonists, including invertebrates^44,47,48^ and by creating a positive association with the forest-forming macroalgae^49–51^. Thus, a reduction of crustose coralline algae cover, because of climate change, may affect the recruitment of forest-forming macroalgae and therefore the maintenance of marine forest habitats^52,53^ and their resistance against climate change^42,54^.

In Mediterranean rocky bottoms, *Cystoseira *sensu lato species (including the genera *Cystoseira*, *Ericaria* and *Gongolaria*, hereafter referred to as *Cystoseira*) are the main representatives of marine forests^13,55^. However, only a few studies have investigated the effect of climate change on this taxon and even fewer have focused on their early stages^21,56–58^. Most studies show a negative impact of OW for both recruits and adults of *Cystoseira* on their survival^26,57,59^, resilience^21^ and phenology^60,61^. In contrast, decreasing pH increased the productivity, antioxidant activity and production of photoprotective compounds of adult *Cystoseira*^56,58^. Despite that, some species of *Cystoseira* (including *Cystoseira compressa* and *Cystoseira foeniculacea*) are considered CCM species whose CCM does not downregulate due to additional CO_2_ and, thus, could not benefit from increasing CO_2_^35^. To our knowledge, there are no studies on the combined effects of OW and OA on early life stages or recruits of *Cystoseira*, and only one study^56^ has investigated the effects of both drivers on adults.

Here, we tested the interactive effects of temperature, pH and species facilitation on the recruitment of *Cystoseira compressa*. This species is a common forest-forming macroalgae that can create dense populations on shallow and sheltered rocky shores around the Mediterranean Sea^62^. It is considered one of the most resistant *Cystoseira* species and it is the only one that is not protected under the Barcelona Convention (Annex II; United Nations Environment Programme/Mediterranean Action Plan-UNEP/MAP)^63^. We designed two separate experiments to test the effects of elevated temperature, low pH and the presence of crustose coralline algae on the early life stages of *C. compressa*. The first experiment focused on the effects of the temperature on the recruits of *C. compressa.* Based on the results of the first experiment, we ran a second complementary experiment to assess the role of the interactive effects of temperature, pH and species facilitation (crustose coralline algae) on the recruitment of *C. compressa.* Because coralline algae are sensitive to OA and are a potentially favourable substrate for the recruitment of *Cystoseira*, we assessed the recruitment of *C. compressa* on living and dead *Neogoniolithon brassica-florida*, one of the most common species in association with shallow *Cystoseira* forests^44,64^. We also compared the recruitment of *Cystoseira* on abiotic artificial clay substrates that have been proposed as an efficient substrate for restoration^59,65,66^. The main hypothesis of this study is that climate change will negatively affect the recruitment of *C. compressa*. Our hypotheses are that OW may have a direct negative impact on the recruits of *C. compressa* while OA may increase their growth and productivity. We also hypothesize that the settlement and survival of *C. compressa* might be indirectly affected by the effects of climate change on its associated understory species (crustose coralline algae) that act as a substrate.

## Results

### In situ temperatures at the donor population location

**Figure 1:**
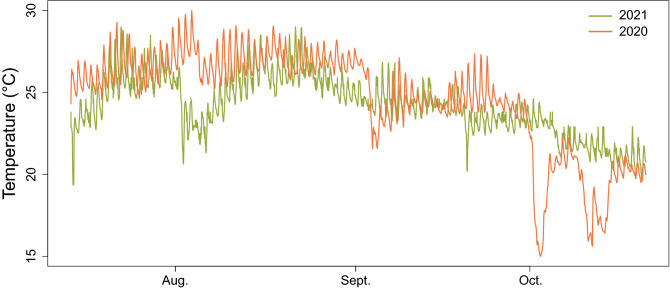
In situ mean seawater temperature at the donor population site.

The temperatures at the *Cystoseira compressa* donor population site during the periods that cover the first (14th July–13th October 2020) and second experiment (10th August–20th October 2021) varied between minimums and maximum values of 15.2 °C (27th October) and 29.5 °C (2nd August) in 2020 and between 19.4 °C (14th July) and 29.0 °C (22nd July) in 2021 (Fig. 1).

### Experiment 1: Effects of ocean warming on the recruitment of *Cystoseira compressa*

**Figure 2:**
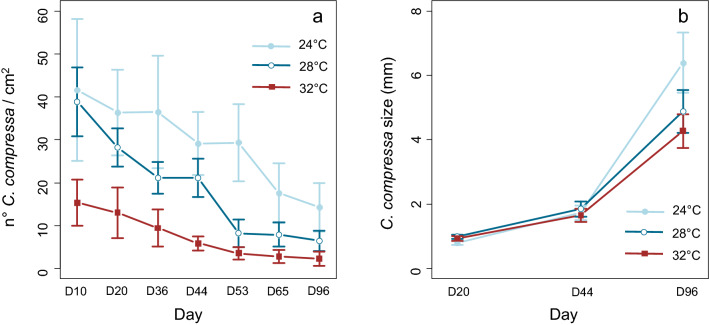
Density (**a**) and size (**b**) of recruits of *C. compressa* as a function of temperature during the first experiment (96 days). The errors bars show the confidence intervals.

The temperature negatively affected the density of recruits since the beginning of the experiment (GLMM, P-value < 0.001; Supplementary material S1). Recruit density was significantly lower at 32 °C than at 28 °C and 24 °C since the first sampling dates (day 10; Fig. 2a), while from day 36 densities at 24 °C remained higher than the ones at 28 °C and 32 °C (Fig. 2a). The temperature also affected the size of the recruits at the end of the experiment, recruits at 24 °C being significantly larger than the ones grown at warmer temperatures (ANOVA, P-value < 0.001; Supplementary material S1 and Fig. 2b).

### Experiment 2: Effects of climate change and species facilitation on the recruitment of *Cystoseira compressa*

#### Recruits of *Cystoseira compressa*

**Figure 3:**
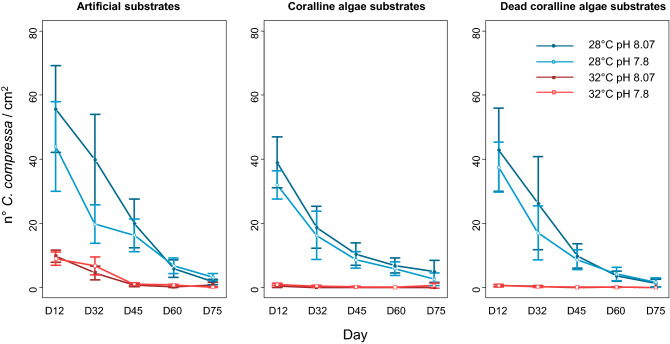
Densities of recruits on living and dead *Neogoniolithon brassica-florida* and on artificial substrates, as a function of temperature and pH under the different treatments. The errors bars show the confidence intervals.

The temperature and pH, separately and together with substrate type and time, had an interactive effect on the density of recruits (GLMM, P-value < 0.001; Supplementary material S2). There was a strong negative effect of the temperature on the density of recruits, especially in presence of *Neogolithon brassica-florida* (Fig. 3). Low pH negatively affected the density of recruits in absence of *N. brassica-florida*, but no differences in density between pH levels were detected in presence of the coralline algae (Supplementary materials S2 and S3). The presence of the living and dead coralline algae negatively affected the density of recruits of *C. compressa*, compared to the higher densities found on artificial substrates. The interaction among temperature, pH and time (Temperature × pH × Time) also affected the density of recruits (GLMM, P-value < 0.001; Supplementary material S2). Higher densities of recruits were found at low temperature-ambient pH while no differences between pH levels were detected at elevated temperature (Supplementary material S2). In general, higher densities of recruits were found at low temperature, ambient pH and in absence of *N. brassica-florida*. The density of recruits decreased with time which led to a homogenization of results for most of the considered factors at the end of the experiment (significant differences in pH and temperature were found only in absence of the coralline algae and at low temperature, Supplementary material S3). This is because only a few recruits survived until the end of the experiment (Fig. 3).

**Figure 4:**
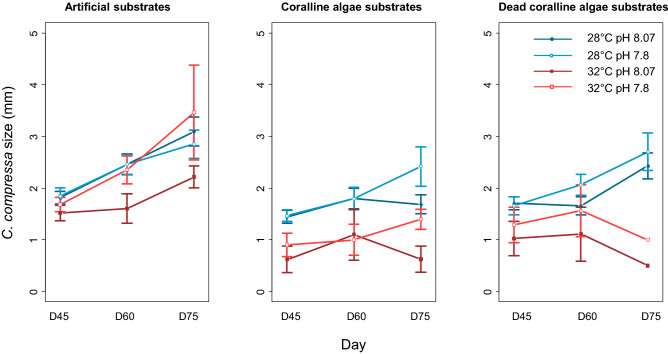
Size of recruits on living and dead *Neogoniolithon brassica-florida* and on artificial substrates as a function of temperature and pH under the different treatments. The errors bars show the confidence intervals.

Several interactive effects involving temperature, pH, presence of coralline algae and time affected the size of recruits (GLMM, P-value < 0.001 for the interactions pH × Substrate Type × Time, Temperature × pH and Temperature × Substrate type, Supplementary material S2). The size of recruits was larger at low temperature and low pH. The size of recruits was also larger in absence of *N. brassica-florida* in all the treatment combinations (Fig. 4). Differences in size were observed between recruits on living and dead *N. brassica-florida*, but only at low temperature. By the end of the experiment, recruits grown in association with the living coralline algae were smaller, while there were no differences in size between recruits grown on dead *N. brassica florida* and in clay substrates (Fig. 4; Supplementary material S4).

#### Calcification of *Neogoniolithon brassica-florida*

**Figure 5:**
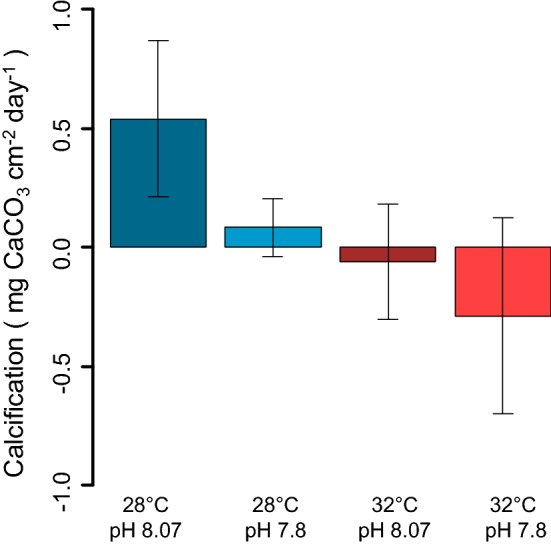
Calcification rate of *Neogoniolithon brassica-florida* after 64 days under different treatments. Error bars show the confidence intervals.

The temperature and pH negatively affected the net calcification of living *Neogoniolithon brassica-florida* (LM, P-value < 0.01 for Temperature and P-value < 0.05 for pH; Supplementary material S2). Calcification rates were statistically significantly higher at 28 °C than at 32 °C and ambient pH than at low pH (Fig. 5).

## Discussion

Marine forests are in regression in many locations worldwide^17,18,67^ and particularly in the Mediterranean Sea^68–70^. Despite this, the response of the different life stages to climate change remains poorly known, efforts have been done in recent years to evaluate the single or combined effect of climate change on forest-forming species together with other local stressors such as pollution, highlighting and strong negative effect of OW^21,71,72^. Our results showed that the temperature, pH and the presence of potentially facilitating species had substantial effects on the density of recruits of *Cystoseira compressa*. The warmer temperature had, in agreement with our initial hypotheses, the largest negative effect on both the density and the size of recruits, as already observed on other forest-forming species, both on recruits^21,26,71^ and adults^28,57,73,74^. Warmer temperatures not only affect the survival and growth of *Cystoseira* but also affect their metabolism^56,75,76^. A study on *C. compressa* reported that the maximum quantum yield (*F*_v_/*F*_m_) of the macroalgae started decreasing from 28 °C, while the total phenolic content increased with seawater temperature^76^. Globally, the increase in seawater temperature is a direct threat to marine forests, isolating forest-forming macroalgae to refuge locations with more suitable conditions, while consequent local extinctions can be expected in the northern limits of distribution of some species (e.g. the northern limit of the Mediterranean Sea)^26,27^. Moreover, indirect temperature-driven effects on marine forests are not negligible, because they contribute to the tropicalization of habitats and the range expansion of warm water species that can re-shape algal communities and trophic cascades^77,78^.

Seawater pH affected both the density and size of recruits in opposite ways, as density was negatively affected by the low pH, whereas size was positively affected. Low pH has been shown to negatively influence the settlement and early life stages of other key species such as the giant kelp^25,79^, corals and molluscs^38^ which has led to the paradigm that early life stages could be more sensitive to global change and therefore could constitute a bottleneck^21,57^. However, in our experiment, lower pH levels positively affected the size of *C. compressa,* potentially showing a better performance as reported in some studies for the giant kelp^80,81^. Non-calcifying macroalgae are generally considered as not particularly sensitive to OA as they can benefit from increasing dissolved CO_2_, particularly the carbon-limited species that do not possess a CCM^6,36^. The increase in dissolved CO_2_ with OA is therefore expected to positively affect carbon-limited species and neutrally or positively affect non-carbon-limited species with CCM^36^. Most large brown macroalgal species have attributes suggesting the presence of CCM, and yet they benefit from increased CO_2_^56,75,82^. The same seems to happen here with the beneficial effects of high CO_2_ on the size of the recruits. *C. compressa*, possessing a high affinity CCM for dissolved inorganic carbon (DIC), would not change the activity of the CCM due to OA and, thus, would not specially benefit under elevated CO_2_. As a result, they would potentially end up being less competitive than other species that will benefit from elevated CO_2_^35^_._ It is important to note that in some of our experimental conditions (i.e. 32 °C and low pH), the biggest sizes corresponded to the lowest densities, which does not allow us to exclude the effect of density-dependent processes in addition to the effect of CO_2_. OA could also have indirect effects on marine forests, by favouring the increased performance of turfs that are generally carbon-limited, especially in the presence of nutrients (e.g. local nutrient pollution)^6,83^. Turf-forming species are fast-growing and therefore are great space competitors that could limit the recruitment of long-life species such as forest-forming species. As a result, turf-forming species could expand and replace foundation species (e.g. *Cystoseira* *s.l*. species) that are already affected by global warming and other anthropogenic impacts^17,84^. This indirect effect of OA on marine forests might decrease the structural complexity of marine forests, compromising their functioning and promoting regime shifts^6,10,85^.

Our results are in agreement with studies suggesting that OA might have a lesser direct effect on forest-forming macroalgae than warming^38^: the effect of the pH on the density of recruits was likely masked by the strong effect of the temperature. Nevertheless, the highest densities were recorded under low temperature (28 °C) and ambient pH (8.07), while the size was negatively affected by the high temperature (32 °C), but positively affected by a decrease in pH (7.8). Complex interactions of abiotic and biotic factors are well known in natural systems. In our study we initially hypothesized that OW and OA would have affected the early-colonizer coralline algae *Neogoniolithon brassica-florida*, decreasing its potential facilitative effect on *C. compressa* recruitment. The calcification rate of the coralline algae was strongly affected by the temperature and pH, probably explaining the stronger effects of the temperature and pH on recruits growing in association with the coralline algae. However, the presence of both living and dead coralline algae had a negative effect on both the density and the size of the recruits, with the artificial clay substrate being the most favourable. Contrary to what is reported in the literature for other species^44^, the very common coralline alga *N. brassica florida* did not have a facilitating role in the recruitment of the later successional species *C. compressa* in our experiment. Since the very beginning of the experiment fewer *C. compressa* were observed in association with the coralline algae, living and dead. In the case of the living coralline algae substrates, the physiological state of *N. brassica-florida* likely does not explain this result as they exhibited calcification rates consistent with those reported in other species of coralline algae^86,87^. The lower recruitment on living coralline could be due to an inhibition of the settlement and development of recruits of *C. compressa* caused by changes in pH or other chemical parameters in the boundary layer formed on the surface of the coralline algae^86,88^. Furthermore, crustose coralline algae have biotic interactions linked to their microbiome^89^ and to their physical and chemical anti-fouling mechanisms to control epiphytes^46,90,91^. These characteristics of the surface of coralline algae could not be optimal for the recruitment of *C. compressa* and hence have reduced the settlement in our experiment. From an applicative point of view, it is worth noting that clay substrate, which is already used in many restoration actions in the Mediterranean Sea^59,65,66^, was an adequate substrate for the settlement of *C*. *compressa*. Our results support that this substrate is of particular interest because it favours settlement and offers many technical practicalities (e.g. they are cheap, biodegradable, easy to produce and can be formed into any shape)^59,65,66^.

In general, our results show that OW and OA additively, affect both the recruitment of *C. compressa* and the calcification rate of *N. brassica-florida.* Interestingly the interactive effect of OW and OA are likely exacerbated in presence of the coralline *N. brassica-florida*. This could be (1) because of the lower recruitment in the presence of coralline algae or (2) because the effects of OW and OA on coralline algae exacerbate, in turn, its inhibiting effect on *C. compressa*^48,53^. Our experiment shows that complex interactions of biotic and abiotic factors could affect the key species that shape marine forest communities, with an ultimate effect at the ecosystem level^24^. As most experimental studies performed in controlled conditions, some limitations have to be highlighted. First of all, our experiment did not allow to separate the effects of the different drivers on the settlement process and the survival of recruits. Secondarily, epiphytes (turf algae) progressively appeared in our experimental tanks and their proliferation was enhanced at higher temperatures and low pH (author's personal observation), which in turn eventually affected the performance of *C. compressa*. But this phenomenon is likely to be observed also in natural conditions, where turf-forming species are expected to proliferate under OW and OA^17^. Many studies on recruits and adults of *Cystoseira* report elevated mortality under experimental conditions, which confirm the difficulty of maintaining *Cystoseira* in tanks^30,59,65^ and of replicating the conditions of natural habitats in the laboratory. This could be due to different variability in light, temperature and flow conditions in the aquarium facilities^22^. Different associated organisms (e.g. microbiome, epiphytic algae, invertebrates) could also explain the different performances observed when culturing these algae^92^. Our experiments were stopped when recruits showed signs of degradation, but the duration of our experiments is consistent with other studies on *Cystoseira*^26,30,57^. The densities obtained in our experiments are extremely high, reaching an average of 94 100.07 ± 89 324.78 *C. compressa* ind. m^−2^ (MEAN ± SD, n = 28) after 2 months in the first experiment and 14 213.20 ± 17.67 *C. compressa* ind. m^−2^ (MEAN ± SD, n = 197) after 2 months in the second experiment (min.: 1000 ind. m^−2^; max.: 320,000 ind. m^−2^ for the first experiment and min.: 10,000 ind. m^−2^ and max.: 210,000 ind. m^−2^ for the second). Interestingly, a parallel study performed by our team at the same time as the first experiment, seedling natural stones with the same technique presented in this study directly in the field, produced lower densities after 2 months. On the contrary, the recruits growing in the field under natural conditions were bigger (11.33 ± 3.27 mm) than in the first (5.18 ± 2.70 mm) and second experiment (2.51 ± 1.61 mm) after 2 months, showing that the conditions in tanks are not optimal for the growth of *C. compressa* and/or that density-dependent factors can result in high density and smaller size of recruits in the laboratory. Natural densities observed in the donor population reach only 76.36 ± 0.72 of *C. compressa* ind. m^−2^ (MEAN ± SD, n = 22; maximum: 128 ind. m^−2^ and minimum: 16 ind./m^−2^; author’s personal observations, article in prep.). Even if the comparison with natural conditions often highlight some limitation for the studies in tanks, it is important to continue with this approach as it is the only one that allows testing multiple factors under controlled conditions (i.e. temperature and pH).

The results from this study demonstrate that the interactive effects of climate change have a pronounced negative impact on shallow marine forests. This result is especially striking for forest-forming macroalgae thriving in rock pools, which are expected to be more acclimated to local factors variation (i.e. temperature and pH). Marine forests are facing several other stressors than global change (e.g. water pollution, urbanization, trampling, herbivory)^19^ that already put them on the edge. Some of these stressors have been addressed by management measures and are now mitigated (e.g. water quality)^93^ allowing, in a few cases, the natural recovery of the forest or the feasibility of planning restoration actions^94–97^. Recently, new restoration techniques opened the door to the possibility of restoring these ecosystems, still, marine forests will be increasingly impacted by global change^1^ and it has to be considered to achieve long-term population sustainability and/or successful restoration actions. The next step for the protection of these key ecosystems is to understand how climate change and other drivers acting at the local scale can interact, eventually providing additive or synergetic effects^98^, likely causing the restructure and redistribution of marine forests and affecting their ability to resist and recover under extreme conditions^24,26,28^.

## Materials and methods

### Experiment 1: Effects of ocean warming on the recruitment of *Cystoseira compressa*

#### Collection and obtention of recruits

Apical fertile branches of *Cystoseira compressa* were hand-collected on the 13th of July 2020 from a donor population situated in a rockpool (between the surface and 1 m depth) in Sainte Marguerite Island (Lérins Islands, France). This site is a Nature 2000 site situated in front of the coast of Cannes and it is one of the last locations with healthy *Cystoseira* populations in the French Riviera^69^. After visually checking that the receptacles contained fertile conceptacles, about 140 g fresh weight (FW) of apical fertile branches were manually collected and transported in cool and dark conditions in plastic bags to the laboratory. The sampling was non-destructive, as only apical branches (roughly 5 cm long) were collected. A temperature data logger (HOBO Pendant MX Temp, ONSET), that took measurements every hour, was installed in the rock pool to monitor the temperature in the donor population site. The receptacles were conserved at 4 °C in the dark overnight before placing them in experimental tanks filled with filtered seawater (20 µm) and marble substrates that acted as settlement substrates^59,66,99^. In each tank, 15 g FW of receptacles were placed in a net on the surface allowing the zygotes to fall on the substrates. The receptacles were kept in the tanks for 72 h without water circulation to facilitate the settlement of the zygotes. The temperature was maintained during the releasing and the settlement of the zygotes at the target temperature treatment. After opening the water system, the receptacles were removed and the recruits were kept in tanks for 96 days. Submersible water pumps (NEWA) provided water motion in each experimental tank.

#### Experimental set-up and treatments

Three independent 5 L tanks (n = 3) were set up for each temperature (24, 28 and 32 °C; Table 1), for a total of 9 experimental tanks, with three square marble substrates of about 25 cm^2^ placed inside each tank. Filtered seawater (20 µm) pumped from Villefranche Bay at 3 m depth was continuously delivered into the experimental tanks at a rate of 7 L h^−1^. The experimental tanks were placed inside a thermoregulated bath to maintain the temperature at the targeted value. The temperature was controlled in two thermoregulated baths per treatment with a temperature controller (T CONTROLLER TWIN AQUA MEDIC). Light was provided by 37 W LED light bars (PRO^2^ LED, Aquaristik) and the irradiance gradually increased from 0 μmol photons m^−2^ s^−1^ at 06:30 to a maximum of 110 μmol photons m^−2^ s^−1^ between 12:00 and 14:00, and gradually decreased to 0 μmol photons m^−2^ s^−1^ at 21:00 (LI-185B with an LI-190SB quantum sensor, LI-COR Biosciences, Lincoln, USA). The three temperature treatments were selected according to the temperatures registered in the donor population during the reproductive season of *C. compressa* and the expected increase in temperature due to global warming.

**Table 1:** Measured (regular characters) and expected (bold characters) seawater physico-chemical parameters (temperature in °C, *p*H_T_ in total scale, calculated *p*CO_2_ in µatm, and total alkalinity in µmol kg^−1^ with MEAN ± SD) according to different treatments.

Treatment	Temperature (°C)	*p*H_T_ n = 15	*p*CO_2_ (µatm) n = 15	Total alkalinity (µmol kg^−1^) n = 8
**24 °C**	24.15 ± 0.43 n = 23	–	–	–
**28 °C**	28.3 ± 0.41 n = 23	–	–	–
**32 °C**	31.47 ± 0.60 n = 23	–	–	–
**28 °C pH 8.07**	28.22 ± 0.71 n = 15	8.04 ± 0.04	434.23 ± 42.63	2564.32 ± 5.80
**28 °C pH 7.8**	28.37 ± 0.74 n = 15	7.83 ± 0.12	795.89 ± 170.97	2568.73 ± 22.51
**32 °C pH 8.07**	31.56 ± 0.47 n = 15	8.01 ± 0.03	468.05 ± 45.53	2564.96 ± 6.23
**32 °C pH 7.8**	31.61 ± 0.70 n = 15	7.83 ± 0.063	788.17 ± 135.69	2563.26 ± 5.50

#### Measurements

The density and size of *C. compressa* recruits were selected as response variables. The density of recruits was calculated by taking pictures every 10 or 15 days and counting from the picture the total number of recruits on a 3 × 3 cm area in the middle of each substrate using the software ImageJ (ImageJ, NIH US Department of Health and Human Services). The size of recruits (µm) was determined monthly on 5 recruits that were removed from each substrate (n = 45). Their total length was measured using a microscope equipped with a graduated eyepiece.

### Experiment 2: Effects of climate change and species facilitation on the recruitment of *Cystoseira compressa*

#### Collection and obtention of recruits

Apical fertile branches of *C. compressa* were collected on the 4th of August 2021 from the same donor population as mentioned above and following the same protocol (see “Collection and obtention of recruits” section from Experiment 1). For this experiment, about 500 g FW of apical fertile branches were manually collected and transported in cool and dark conditions in plastic bags to the laboratory. The receptacles were conserved at 4 °C in the dark overnight before placing them under the experimental conditions, in the experimental tanks, to obtain recruits on the different substrates and under the different conditions of temperature and acidification. In each tank, 10 g FW of receptacles placed inside a net were kept on the surface for 72 h. During this period, the temperature and pH were maintained at the target treatment conditions. Afterwards, the receptacles were removed and the recruits were kept in tanks for 75 days.

#### Experimental set-up and treatments

The recruitment of *Cystoseira compressa* was assessed in two different conditions of temperature, 28 and 32 °C, and two pH levels, ambient (pH_T_ = 8.07) and low pH (pH_T_ = 7.8). In order to investigate the potential facilitation effect of *Neogoniolithon brassica-florida*, three different substrates were used for the settlement of recruits: (1) living and (2) dead *N. brassica-florida* (respectively factors “coralline” and “dead coralline”) and (3) artificial clay substrates (factor “artificial substrate”). The temperatures were selected according to the results obtained during the first experiment (experiment 1). The low pH condition (pH_T_ = 7.8) corresponds to the pH value expected by the end of the century under the SSP2—4.5 CO_2_ emissions scenario^75,100^. The surface of the three substrates was about 10 cm^2^ (3 cm diameter and 1 cm height for the artificial substrates). The coralline algae were collected in April 2021 from Anse des Fossés (Saint-Jean-Cap-Ferrat, France) between 0.5 and 1 m depth. Samples that were the most homogeneous and the least colonized by other organisms were selected. Then, they were cleaned and epiphytes were removed using brushes and tweezers. Dead coralline substrates were obtained by putting the *N. brassica-florida* substrates in freshwater with bleach (1:50) for 24 h. They were then rinsed several times with freshwater and dried, before placing them in the tanks. All the substrates were placed in the experimental tanks at ambient seawater temperature and pH and gradually brought to the experimental temperatures and pH levels over 3 months before the start of the experiment.

**Figure 6:**
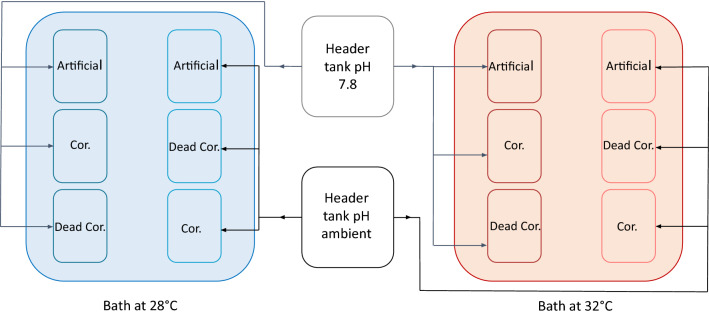
Experimental set-up used to test the effects of temperature (28 and 32 °C), pH (ambient 8.07, and low 7.8) and species facilitation (artificial, coralline and dead coralline substrates) on the recruits of *C. compressa*. The experimental set-up was repeated 4 times, resulting in 8 header tanks, and 48 experimental tanks in which the different types of substrates were randomly assigned.

The experimental set-up consisted of four independent 1.8 L tanks (n = 4) for each of the 12 conditions (Substrate type × pH × Temperature) for a total of 48 experimental tanks. Each tank contained five replicates of a substrate (either artificial, coralline or dead coralline substrates). One substrate from each experimental tank was used as a control and was not seeded with zygotes of *C. compressa*. Seawater from the Bay of Villefranche was continuously delivered into eight 20 L header tanks that then gravity fed six 1.8 L independent experimental tanks each with a water rate of 3 L h^−1^ (Fig. 6). Seawater pH was manipulated inside 4 20 L header tanks. pH was maintained at the target value using pH controllers (APEX, Neptune Systems) that controlled the bubbling of pure CO_2_ in the header tanks. The experimental tanks were placed inside thermoregulated baths (four per temperature) connected to the same control system (APEX, Neptune Systems; Fig. 6) to maintain the temperature at 28 and 32 °C. Submersible water pumps (NEWA) provided water motion in each experimental tank.

Light was provided by 89 W LED light bars (Aqualumix, Aquaristik) and irradiance gradually increased from 0 μmol photons m^−2^ s^−1^ at 06:30 to a maximum of 175 μmol photons m^−2^ s^−1^ between 12:00 and 14:00, and gradually decreased to 0 μmol photons m^−2^ s^−1^ at 21:00 (LI-185B with an LI-190SB quantum sensor, LI-COR Biosciences, Lincoln, USA)^26^.

#### Carbonate chemistry

pH in the header and experimental tanks was measured weekly using a handheld pH-meter (826 pH mobile, Metrohm) calibrated with TRIS buffer (batch #T33 provided by A. Dickson, Scripps Institution of Oceanography). Total alkalinity was measured weekly in eight randomly selected tanks and was determined by potentiometric titration using a Metrohm 888 Titrando following the method of Dickson et al.^101^, the samples were measured three times and the mean value was used. Certified reference material (Batch #186) provided by A. Dickson was used to assess the accuracy of the measurements and was within 7.73 µmol kg^−1^. The seawater from the Bay of Villefranche was 2565.41 ± 12.1 μmol kg^−1^ (MEAN ± SD; Table 1; Supplementary material S5).

#### Measurements

The density and size of recruits of *C. compressa* and calcification rate of the living coralline algae substrates were selected as response variables. The density of recruits was assessed by counting directly in the tanks the total number of recruits on the substrates using a magnifying table lamp. The total number of recruits was normalized by the surface of the substrate. The size of the recruits was determined by measuring the length of ten individuals randomly picked from each substrate (n = 160). When ten or fewer individuals were present on the substrate, all of them were measured. The measurements were done using graph paper under a magnifying table lamp.

Total calcification rate of *N. brassica-florida* was assessed using the buoyant weight technique^102^. Weighing was done before obtaining *C. compressa* recruits and at the end of the experiment, 64 days later. Changes in wet weight were converted to dry weight using the following equation:

$$Dry \; weight = \frac{Wet \; weight}{\left(1 - \frac{Water\; density}{Calcite \;density}\right)}$$

with a calcite density of 2.73 g cm^−3^. Calcification rate was determined as the change in dry weight normalized by the surface of coralline algae at the moment of the weighting and the number of days (64 days). Surfaces of coralline substrates were determined on photographs using the software ImageJ (ImageJ, NIH US Department of Health and Human Services).

All experiments were carried out according to relevant regulations and guidelines concerning *Cystoseira compressa* and *Neogoniolithon brassica-florida* sampling. The latter were collected under prefectoral order No. 277, delivered by the Interregional Directorate of the Mediterranean Sea, Regulatory/Control Service, authorizing the ECOSEAS Laboratory to sample fauna and flora for scientific purposes only.

### Data analysis

#### Experiment 1

A Generalized Linear Mixed-Effects Model (GLMM), with a Poisson link log distribution function was used to test the effect of the temperature on the density of recruits, with temperature (three levels) and time (seven levels) as fixed factors, and substrate nested within tank as random. A two-way ANOVA was used to test the effect of temperature on the size of the recruits, with temperature (three levels) and time (three levels) as fixed factors. The assumptions of normality and equality of variance were evaluated through graphical analyses of residuals using QQ plot functions. An alpha of 0.01 was used when the assumption of equality of variance was not achieved.

#### Experiment 2

A GLMM with a Poisson distribution was used to test the effect of temperature, pH and substrate type on the density of recruits, with temperature (two levels), pH (two levels), substrate type (three levels) and time (five levels) as fixed factors and substrate nested within tanks as random, to account for the lack of independence between observations (repeated measures over time). The response of the variable size to the treatments was analysed using a GLMM with a Gamma error distribution function and the logit link function ‘inverse’, with temperature (two levels), pH (two levels), substrate type (three levels) and time (three levels) as fixed factors and tank as random. The total calcification rate of the coralline algae was analysed with a Linear Model (LM), with temperature (two levels) and pH (two levels) as fixed factors.

GLMM and LM models were fitted to analyse the effect of the variables and the AICs likelihood minimum was used to select the best model among the possible combinations. The different models were fitted using the functions “glmer” and “lm” from the package lme4^103^ in the statistical environment R^104^. P-values were obtained by means of a Wald χ^2^ test using the “ANOVA” function from the CAR package^105^. Finally, the function “emmeans” from the package emmeans^106^ was used to perform the post-hoc analysis of the LM and GLMM models while the test “snk” (Student–Newman–Keuls) was used to perform the post-hoc analysis for the two-way ANOVA.

## Supplementary Information

Supplementary Information.

## Data Availability

The datasets generated and analysed during the current study are available in the Knowledge Network for Biocomplexity repository, https://knb.ecoinformatics.org/view/urn:uuid:daa9cc97-47eb-48a3-addc-ed0047f0f3c4.
