# Supplementary material for: Climate change and species facilitation affect the recruitment of macroalgal marine forests
Source: Sci Rep. 2022 Oct 27;12:18103. doi: 10.1038/s41598-022-22845-2 (PMC9613703; doi:10.1038/s41598-022-22845-2)
Supplement: Supplementary file 1 — Supplementary Information. [file 41598_2022_22845_MOESM1_ESM.pdf]

Climate change and species facilitation affect the recruitment of macroalgal marine forests

Supplementary material S1: Statistic results from the experiment 1.

	Variable	Model	Factor levels	DF		Chi sq		P-value
**Experiment 1**	**C.compressa/cm2**	**glmer (C.compressa/cm2 ~ Temperature*Day + (1|Tank/Substrate), family="poisson", na.action = na.omit)**	Temperature (28°C and 32°C)	2		296.56		<0.01
			Day (7 days)	6		581.09		<0.01
			Temperature*Day	12		42.937		<0.01
	**Variable**	**Model**	**Factor levels**	**DF**	**Sum sq**	**Mean sq**	**F value**	**P-Value**
	**Size C. compressa (mm)**	**Anova (Size C. compressa ~Temperature+Day+Temperature*Day)**	Temperature (28°C and 32°C)	2	1	768590551	316.643	<0.01
			Day (3 days)	2	3	18087101	7.451	<0.01
			Temperature*Day	4	8	21189426	8.730	<0.01
			Residuals	441	1	2427311		

Climate change and species facilitation affect the recruitment of macroalgal marine forests

Supplementary material S2: Statistic results from the experiment 2.

	Variable	Model	Factor levels	DF		Chi sq	P-value
Experiment 2	*C. compressa* /cm2	**glmer (C.compressa/cm^2 ~ Temperature*pH*Substrate Type*Day + (1|Tank/Substrate), family="poisson", na.action = na.omit))**	Temperature (28°C and 32°C)	6		628.25	<0.01
			pH (ambient and 7.8)	1		134.99	<0.01
			Substrate type (artificial, Corallinales and dead Corallinales)	7		145.70	<0.01
			Day (6 days)	9		4362.03	<0.01
			Temperature*pH	1		36.81	<0.01
			Temperature*Substrate type	5		129.45	<0.01
			pH*Substrate type	2		10.38	<0.01
			Temperature*Day	7		107.99	<0.01
			pH*Day	4		40.74	<0.01
			Substrate type * Day	11		157.09	<0.01
			Temperature*pH*Substrate type	2		3.94	>0.05
			Temperature*pH*Day	4		41.87	<0.01
			Temperature*Substrate type*Day	8		30.97	<0.01
			pH*Substrate type*Day	8		45.99	<0.01
			Temperature*pH*Substrate type*Day	8		1.28	>0.05
	Size *C. compress* a (mm)	**glmer (C. compressa size ~ Temperature*pH*Substrate type*Day + (1|Individual), Gamma(link = "inverse"), na.action = na.omit=subs)**	Temperature (28°C and 32°C)	1		0.59	>0.05
			pH (ambient and 7.8)	1		7.82	<0.01
			Substrate type (artificial, dead Corallinales and Corallinales)	2		193.46	<0.01
			Day	2		277.65	<0.01
			Temperature*pH	1		16.31	<0.01
			Temperature*Substrate type	2		66.18	<0.01
			pH*Substrate type	2		17.92	<0.01
			Temperature*Day	2		2.20	>0.05
			pH*Day	2		4.40	>0.05
			Substrate type*Day	4		8.80	>0.05
			Temperature*pH*Substrate type	2		1.83	>0.05
			Temperature*pH*Day	2		1.96	>0.05
			Temperature*Substrate type*Day	4		3.26	>0.05
			pH*Substrate type*Day	4		19.12	<0.01
			Temperature*pH*Substrate type*Day	4		5.08	>0.05
	**Variable**	**Model**	**Factor levels**	**DF**	**Sum sq**	**F value**	**P-Value**
	**Calcification rate (mg CaCO3 cm-2 day-1)**	**lm (Calcification rate ~ Temperature*pH, na.action = na.omit)**	Temperature (28°C and 32°C)	1	7.38	8.6446	<0.01
			pH (ambient and 7.8)	1	3.96	4.6321	<0.05
			Temperature*pH	1	0.15	0.1789	>0.05
			Residuals	118	100.80		

Climate change and species facilitation affect the recruitment of macroalgal marine forests

Supplementary material S3: Results from the post-hoc analysis of the experiment 2 on the density of *C. compressa* for the significant interactions pH x Substrate Type x Day, Temperature x Substrate Type x Day and Temperature x pH x Day. AS is artificial substrates, CS coralline algae substrates and DCS dead coralline algae substrates.

(*) significant differences (=) no significant differences			pH ambient															pH 7.8														
			AS	AS	AS	AS	AS	DCS	DCS	DCS	DCS	DCS	CS	CS	CS	CS	CS	AS	AS	AS	AS	AS	DCS	DCS	DCS	DCS	DCS	CS	CS	CS	CS	CS
			D12	D32	D45	D60	D75	D12	D32	D45	D60	D75	D12	D32	D45	D60	D75	D12	D32	D45	D60	D75	D12	D32	D45	D60	D75	D12	D32	D45	D60	D75
pH ambient	AS	D12		*	*	*	*	*					*					*														
pH ambient	AS	D32	*		*	*	*		*					=					*													
pH ambient	AS	D45	*	*		*	*			=					=					=												
pH ambient	AS	D60	*	*	*		=				=					=					*											
pH ambient	AS	D75	*	*	*	=						=					=					*										
pH ambient	DCS	D12	*						=	=	*	=	=										=									
pH ambient	DCS	D32		*				=		=	=	*		=										=								
pH ambient	DCS	D45			=			=	=		=	=			=										=							
pH ambient	DCS	D60				=		*	=	=		=				=										=						
pH ambient	DCS	D75					=	=	*	=	=						=										=					
pH ambient	CS	D12	*					=						=	=	=	=											=				
pH ambient	CS	D32		=					=				=		=	=	=												=			
pH ambient	CS	D45			=					=			=	=		=	=													=		
pH ambient	CS	D60				=					=		=	=	=		=														=	
pH ambient	CS	D75					=					=	=	=	=	=																=
pH 7.8	AS	D12	*																*	*	*	*	*					*				
pH 7.8	AS	D32		*														*		*	*	*		*					*			
pH 7.8	AS	D45			=													*	*		*	*			*					*		
pH 7.8	AS	D60				*												*	*	*		*				*					*	
pH 7.8	AS	D75					*											*	*	*	*						=					=
pH 7.8	DCS	D12						=										*						=	*	*	=	=				
pH 7.8	DCS	D32							=										*				=		*	=	=		=			
pH 7.8	DCS	D45								=										*			*	*		=	=			=		
pH 7.8	DCS	D60									=										*		*	=	=		=				=	
pH 7.8	DCS	D75										=										=	=	=	=	=						=
pH 7.8	CS	D12											=					*					=						*	*	*	*
pH 7.8	CS	D32												=					*					=				*		=	=	=
pH 7.8	CS	D45													=					*					=			*	=		=	=
pH 7.8	CS	D60														=					*					=		*	=	=		=
pH 7.8	CS	D75															=					=					=	*	=	=	=	

Climate change and species facilitation affect the recruitment of macroalgal marine forests

			28°C															32°C														
			AS	AS	AS	AS	AS	DCS	DCS	DCS	DCS	DCS	CS	CS	CS	CS	CS	AS	AS	AS	AS	AS	DCS	DCS	DCS	DCS	DCS	CS	CS	CS	CS	CS
			D12	D32	D45	D60	D75	D12	D32	D45	D60	D75	D12	D32	D45	D60	D75	D12	D32	D45	D60	D75	D12	D32	D45	D60	D75	D12	D32	D45	D60	D75
28°C	AS	D12		*	*	*	*	=					*					*														
	AS	D32	*		*	*	*		*					*					*													
	AS	D45	*	*		*	*			*					*					*												
	AS	D60	*	*	*		*				*					=					*											
	AS	D75	*	*	*	*						*					=					*										
	DCS	D12	=						*	*	*	*	=										*									
	DCS	D32		*				*		*	*	*		=										*								
	DCS	D45			*			*	*		*	*			=										=							
	DCS	D60				*		*	*	*		*				*										*						
	DCS	D75					*	*	*	*	*						*										=					
	CS	D12	*					=						*	*	*	*											*				
	CS	D32		*					=				*		*	*	*												=			
	CS	D45			*					=			*	*		*	*													=		
	CS	D60				=					*		*	*	*		*														=	
	CS	D75					=					*	*	*	*	*																=
32°C	AS	D12	*																*	*	*	*	*					*				
	AS	D32		*														*		*	*	*		*					=			
	AS	D45			*													*	*		=	=			=					=		
	AS	D60				*												*	*	=		=				*					=	
	AS	D75					*											*	*	=	=						=					=
	DCS	D12						*										*						=	=	*	=	=				
	DCS	D32							*										*				=		=	=	=		=			
	DCS	D45								=										=			=	=		=	=			=		
	DCS	D60									*										*		*	=	=		=				=	
	DCS	D75										=										=	=	=	=	=						=
	CS	D12											*					*					=						=	=	=	=
	CS	D32												=					=					=				=		=	=	=
	CS	D45													=					=					=			=	=		=	=
	CS	D60														=					=					=		=	=	=		=
	CS	D75															=					=					=	=	=	=	=	

			pH ambient 28°C					pH 7.8 28°C					pH ambient 32°C					pH 7.8 32°C				
			D12	D32	D45	D60	D75	D12	D32	D45	D60	D75	D12	D32	D45	D60	D75	D12	D32	D45	D60	D75
pH ambient	28°C	D12		*	*	*	*	*					*									
		D32	*		*	*	*		*					=								
		D45	*	*		*	*			*					=							
		D60	*	*	*		*				=					=						
		D75	*	*	*	*						=					=					
pH 7.8	28°C	D12	*						*	*	*	*						*				
		D32		*				*		*	*	*							*			
		D45			*			*	*		*	*								*		
		D60				=		*	*	*		*									*	
		D75					=	*	*	*	*											=
pH ambient	32°C	D12	*											=	=	=	=	=				
		D32		=									=		=	=	=		=			
		D45			=								=	=		=	=			=		
		D60				=							=	=	=		=				=	
		D75					=						=	=	=	=						=
pH 7.8	32°C	D12						*					=						=	*	*	=
		D32							*					=				=		*	*	=
		D45								*					=			*	*		=	=
		D60									*					=		*	*	=		=
		D75										=					=	=	=	=	=	

Climate change and species facilitation affect the recruitment of macroalgal marine forests

Supplementary material S4: Results of the post-hoc analysis of the experiment 2 on the size of *C. compressa* for the significant interactions pH x Substrate Type x Day, Temperature x Substrate Type and Temperature x pH. AS is artificial substrates, CS coralline algae substrates and DCS dead coralline algae substrates.

(*) significant differences (=) no significant differences			pH ambient									pH 7,8								
			AS D45	AS D60	AS D75	DCS D45	DCS D60	DCS D75	CS D45	CS D60	CS D75	AS D45	AS D60	AS D75	DCS D45	DCS D60	DCS D75	CS D45	CS D60	CS D75
pH ambient	AS	D45		=	*	*			*	*		=								
	AS	D60	=		*		*						*							
	AS	D75	*	*				=			*			=						
	DCS	D45	*				=	=	*						=					
	DCS	D60		*		=		=		=						=				
	DCS	D75			=	=	=				=						=			
	CS	D45	*			*				*	=							=		
	CS	D60		*			=		*		=								=	
	CS	D75			*			=	=	=										*
pH 7,8	AS	D45	=										*	*	*			*		
	AS	D60		*								*		*		*			*	
	AS	D75			=							*	*				=			*
	DCS	D45				=						*				=	=	=		
	DCS	D60					=						*		=		=		=	
	DCS	D75						=						=	=	=				
	CS	D45							=			*			=	=			=	*
	CS	D60								=			*					=		=
	CS	D75									*			*			=	*	=	

		28°C			32°C		
		AS	DCS	CS	AS	DCS	CS
28°C	AS		*	*	=		
	DCS	*		*		*	
	CS	*	*				*
32°C	AS	=				*	*
	DCS		*		*		=
	CS			*	*	=	

		pH ambient		pH 7.8	
		28°C	32°C	28°C	32°C
pH ambient	28°C		*	*	
	32°C	*			*
pH low	28°C	*			*
	32°C		*	*	

Supplementary material S5: Measured (regular characters) and expected (bold characters) seawater physico-chemical parameters (temperature in °C, *p*HT in total scale, calculated pCO_2 in µatm, and total alkalinity in µmol Kg $^{-1}$ with MEAN and SD) according to different treatments and substrate types.

Treatment	Substrate type	Temperature (°C)		pH		$p\ CO_2$ (µatm)		Total alkalinity (µmol kg^{-1})	
		Mean	SD	Mean	SD	Mean	SD	Mean	SD
**28°C pH 8.07**	**Artificial**	28,25	0,79	8,05	0,05	429,42	57,51	2562,06	5,79
	**Coralline**	28,19	0,66	8,04	0,04	439,46	50,41	2563,77	3,60
	**Dead Coralline**	28,21	0,69	8,04	0,04	435,52	50,13	2566,66	7,20
**28°C pH 7.8**	**Artificial**	28,29	0,75	7,83	0,14	807,15	192,03	2564,20	3,52
	**Coralline**	28,38	0,73	7,84	0,07	784,99	154,70	2564,79	15,41
	**Dead Coralline**	28,43	0,73	7,84	0,13	793,41	166,83	2561,56	3,59
**32°C pH 8.07**	**Artificial**	31,57	0,44	8,01	0,04	468,46	47,74	2566,97	5,36
	**Coralline**	31,58	0,48	8,00	0,03	477,15	39,59	2565,73	6,77
	**Dead Coralline**	31,56	0,48	8,02	0,04	459,05	47,13	2562,03	6,51
**32°C pH 7.8**	**Artificial**	31,61	0,76	7,82	0,07	805,88	155,87	2561,92	4,89
	**Coralline**	31,54	0,70	7,83	0,06	783,92	135,27	2562,58	6,06
	**Dead Coralline**	31,67	0,64	7,84	0,05	774,72	112,88	2565,43	5,90
